# Assessment of metal-assisted nucleophile activation in the hepatitis delta virus ribozyme from molecular simulation and 3D-RISM

**DOI:** 10.1261/rna.051466.115

**Published:** 2015-09

**Authors:** Brian K. Radak, Tai-Sung Lee, Michael E. Harris, Darrin M. York

**Affiliations:** 1 Center for Integrative Proteomics Research and Department of Chemistry and Chemical Biology, Rutgers University, Piscataway, New Jersey 08854-8076, USA; 2 Department of Chemistry, University of Minnesota, Minneapolis, Minnesota 55455-0431, USA; 3 Department of Biochemistry, Case Western Reserve University School of Medicine, Cleveland, Ohio 44106, USA

**Keywords:** QM/MM, 3D-RISM, RNA catalysis, mechanism, free energy

## Abstract

The hepatitis delta virus ribozyme is an efficient catalyst of RNA 2′-*O*-transphosphorylation and has emerged as a key experimental system for identifying and characterizing fundamental features of RNA catalysis. Recent structural and biochemical data have led to a proposed mechanistic model whereby an active site Mg^2+^ ion facilitates deprotonation of the O2′ nucleophile, and a protonated cytosine residue (C75) acts as an acid to donate a proton to the O5′ leaving group as noted in a previous study. This model assumes that the active site Mg^2+^ ion forms an inner-sphere coordination with the O2′ nucleophile and a nonbridging oxygen of the scissile phosphate. These contacts, however, are not fully resolved in the crystal structure, and biochemical data are not able to unambiguously exclude other mechanistic models. In order to explore the feasibility of this model, we exhaustively mapped the free energy surfaces with different active site ion occupancies via quantum mechanical/molecular mechanical (QM/MM) simulations. We further incorporate a three-dimensional reference interaction site model for the solvated ion atmosphere that allows these calculations to consider not only the rate associated with the chemical steps, but also the probability of observing the system in the presumed active state with the Mg^2+^ ion bound. The QM/MM results predict that a pathway involving metal-assisted nucleophile activation is feasible based on the rate-controlling transition state barrier departing from the presumed metal-bound active state. However, QM/MM results for a similar pathway in the absence of Mg^2+^ are not consistent with experimental data, suggesting that a structural model in which the crystallographically determined Mg^2+^ is simply replaced with Na^+^ is likely incorrect. It should be emphasized, however, that these results hinge upon the assumption of the validity of the presumed Mg^2+^-bound starting state, which has not yet been definitively verified experimentally, nor explored in depth computationally. Thus, further experimental and theoretical study is needed such that a consensus view of the catalytic mechanism emerges.

## INTRODUCTION

The hepatitis delta virus ribozyme (HDVr) is a small, self-cleaving ribozyme found in the genome of a satellite of the hepatitis B virus (Kuo et al. 1988; Sharmeen et al. 1988). Of the few known viral ribozymes, it is the only one found in an animal virus (Lai 1995) and is of particular interest due to the existence of similar sequences in both the human genome (Salehi-Ashtiani et al. 2006) and the genome of many other eukaryotes (Webb et al. 2009; Webb and Lupták 2011). It is now generally accepted that ribozymes like the HDVr use a variety of catalytic strategies, including site-specific shifts of nucleobase p*K*_a_’s and/or recruitment of divalent metal ions to stabilize electrostatically strained structures (Fedor 2009; Schnabl and Sigel 2010; Wong and Pollack 2010; Golden et al. 2013; Ward et al. 2014; Panteva et al. 2015). These motifs are well established from detailed experimental and theoretical analysis of the hairpin and hammerhead ribozymes (Lee et al. 2007, 2009; Leclerc 2010; Suydam et al. 2010; Cottrell et al. 2011; Kath-Schorr et al. 2012).

Indeed, experimental studies of the HDVr have identified a specific cytosine residue, C75, as being critical for catalysis (Thill et al. 1993; Ferré-D'Amaré et al. 1998; Perrotta et al. 1999; Nakano et al. 2000; Shih and Been 2001) and a broad range of evidence supports a scenario in which the p*K*_a_ of C75 is shifted ∼2 units toward neutrality compared to both a single nucleotide in solution (Gong et al. 2007) and the cleaved product state (Lupták et al. 2001). Furthermore, biochemical data support the supposition that this residue donates a proton to the leaving group, thus acting as an acid catalyst (Das and Piccirilli 2005; Perrotta et al. 2006).

In addition to their role in RNA folding, metal ions also contribute to HDVr catalysis. The HDVr requires millimolar concentrations of Mg^2+^ ions (Wu et al. 1989; Rosenstein and Been 1990; Been et al. 1992)—or some other divalent ion (Suh et al. 1993)—in order to reach an optimal reaction rate under near-physiological conditions. Detailed biochemical and kinetic studies have revealed multiple functional divalent metal binding sites and demonstrated that molar concentrations of monovalent ions alone can support catalysis (Nakano et al. 2001, 2003; Perrotta and Been 2006). Site-bound metal ion interactions have been identified and characterized via crystallography (Ke et al. 2004, 2007; Chen et al. 2010), spectroscopy (Gong et al. 2009), chemical probing experiments (Chen et al. 2009, 2013; Lévesque et al. 2012; Thaplyal et al. 2015), and molecular simulation (Lee et al. 2011; Veeraraghavan et al. 2011a,b; Chen et al. 2013). Moreover, pH-rate profiles for the reaction in the absence of Mg^2+^ and for mutants designed to disrupt binding of the proposed active site ion are inverted relative to the reaction of the native HDVr in Mg^2+^ (Perrotta and Been 2006; Chen et al. 2013). Phosphorothioate interference studies have revealed sites of potential site-bound metal ion interactions via coordination to one or more nonbridging oxygens, including the *pro*-R_P_ position of the scissile phosphate (Jeoung et al. 1994; Fauzi et al. 1997; Prabhu et al. 1997; Raines and Gottlieb 1998; Oyelere et al. 2002; Das and Piccirilli 2005; Perrotta and Been 2006; Thaplyal et al. 2013). Thiophilic metal ion rescue experiments have been interpreted as supporting a catalytic metal ion interacting with this position. However, unlike typical results for other metalloribozymes (Frederiksen and Piccirilli 2009), the metal rescue is unconventional in that the substitution decreases the susceptibility of the ribozyme to inhibition by thiophilic metal ions rather than being activated by their presence (Thaplyal et al. 2013). Recent studies of stereospecific thio effects over a wide range of ionic conditions reveal inverse thio effects at the *pro*-S_P_ position with large monovalent and divalent ions (Thaplyal et al. 2015). These results, taken together with pH-rate profiles, proton inventories, ammonium/imidazole rescue experiments, and computational simulations, have been interpreted to suggest that the HDVr mechanism can shift from a concerted metal ion-stabilized to a stepwise proton transfer-stabilized pathway with decreasing metal ion charge density (Thaplyal et al. 2015). Clearly the evidence for the participation of an active site metal is very strong, but the details of its interactions in the transition state and its role in catalysis are not yet clear.

Computational simulation allows for direct exploration of specific mechanistic pathways of ribozymes as well as identification of experimental observables that can potentially be used to discriminate between them (Panteva et al. 2015). In the case of the HDVr, molecular simulations can be used to specifically position Mg^2+^ at locations thought to facilitate catalysis. The results of these simulations can then be used to evaluate: (1) the thermodynamics of ion association, (2) the catalytic competency of the resulting configurations, and (3) the structural and dynamical characteristics of mechanistic pathways and how they might affect experimental observables.

**FIGURE 1.**
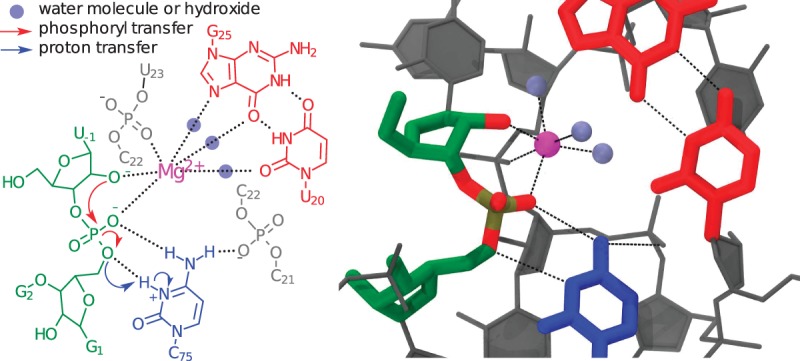
Schematic diagram of the catalytic mechanism proposed by Chen et al. (2010) and Golden (2011). Biochemical, structural, and computational data together indicate that a protonated cytosine residue (blue) acts as an acid to transfer a proton to the O5′ leaving group (blue arrows). Notably, the model shows that a hexacoordinated Mg^2+^ ion (magenta) with three water mediated contacts (gray spheres) to a GU reverse wobble (red) as well as a hydrogen bond between cytosine and the scissile phosphate aid in organizing the active site. The three remaining Mg^2+^ ligands are phosphate oxygens, including the O2′ nucleophile. This interaction is proposed to play a role in activating the nucleophile by lowering its p*K*_a_ to favor the formation of the more reactive O2′ oxyanion.

Recent quantum mechanical/molecular mechanical (QM/MM) studies, both via adiabatic calculations (Ganguly et al. 2011) and the string method (Ganguly et al. 2014; Thaplyal et al. 2015), have focused primarily on analyzing the details of a specific set of active site metal interactions proposed by Chen et al. (2010) and Golden (2011) (Fig. 1). This mechanistic model was inferred from both crystallographic data and structural modeling via homology with the hammerhead ribozyme and is consistent with much of the available biochemical data (Golden 2011). The calculations have provided a detailed catalytic pathway partially consistent with experimental data as well as some specific mechanistic predictions. These simulations predicted a change from a concerted mechanism when the active site Mg^2+^ is present to a stepwise pathway (via a protonated phosphorane intermediate) in the presence of Na^+^ and absence of Mg^2+^ (Ganguly et al. 2014).

The work presented here provides a detailed computational perspective on active site Mg^2+^ association and its contribution to catalysis. This is done by examining the distribution of states connecting divalent metal ion binding and nucleophile activation within a thermodynamic framework based on a molecular mechanics/three-dimensional reference interaction site model (MM/3D-RISM). The states predicted to be catalytically competent are then examined with QM/MM simulations. An overarching theme in this work is that, in order to draw direct comparison with experimental kinetic data, one must consider not only the rate associated with the chemical steps of the reaction departing from a presumed active state with a Mg^2+^ bound in the active site, but also the probability of observing the system in that active state. The QM/MM results suggest that, under the assumption of the validity of the metal ion binding mode described above, a pathway involving metal-assisted nucleophile activation is feasible with a rate constant in reasonable agreement with experiment. However, QM/MM results for a similar pathway in the absence of Mg^2+^ are not in agreement with experiment, suggesting that the structural model in the absence of Mg^2+^ is likely incorrect or, at the very least, incomplete. Further, there remain questions regarding the interpretation of other experimental biochemical data within this mechanistic scenario. These include possible conformational switching of a wobble pair associated with Mg^2+^ binding (Lévesque et al. 2012; Chen et al. 2013), mutational effects that should be related to Mg^2+^ binding but are outside of the proposed active site (Chen et al. 2009; Gong et al. 2009), and anti-correlation of Mg^2+^ binding and the observed p*K*_a_ shift in C75 (Gong et al. 2007).

## RESULTS

### Simulations of the HDVr in different precatalytic states

**FIGURE 2.**
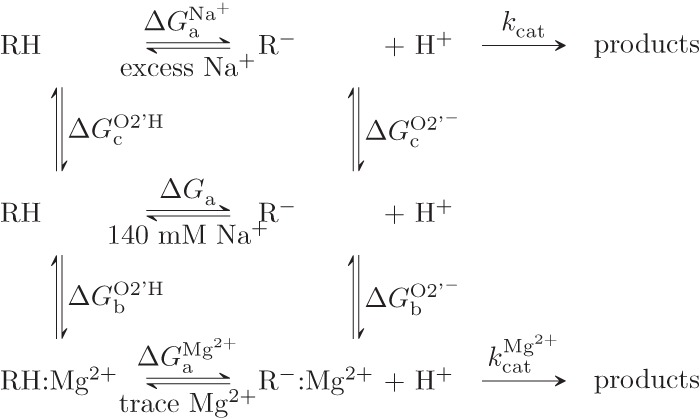
Reaction scheme for (de)protonation of the HDVr at U-1:O2′ and Mg^2+^ binding events needed to attain catalytically active states with and without Mg^2+^ (*k*_cat_^Mg2+^ and *k*_cat_, respectively). RH indicates a (neutral) protonated reactant state (O2′H), while R^−^ indicates a deprotonated reactant state (O2′^−^). In all cases, C75 is assumed to be in a protonated state.

Multiple HDVr states (RH, R^−^, RH:Mg^2+^, and R^−^:Mg^2+^) were defined based on the protonation state of U-1:O2′ and whether or not Mg^2+^ was specifically bound as in the crystallographic model (see Figs. 1, 2; Chen et al. 2010; Golden 2011). As a baseline exploration of the conformational space available to each state, long-time MD trajectories were propagated for at least 100 nsec of data collection. In all states, the trajectories displayed remarkable stability in an active, inline conformation of the U-1:O2′ nucleophile. This was true regardless of whether or not a crystallographically resolved Mg^2+^ ion was artificially ejected from the active site (by swapping coordinates with a bulk Na^+^ ion, as in the RH and R^−^ states) or the nucleophile was protonated or deprotonated (as in the R^−^ and R^−^:Mg^2+^ states). In order to evaluate these conformational searches, cluster analysis was performed and yielded single dominant clusters (>80% occupancy) with small fluctuations of the active site heavy atoms, although this was less true in the case of a deprotonated nucleophile.

### Solvation analysis with 3D-RISM and NLPB-SA

**FIGURE 3.**
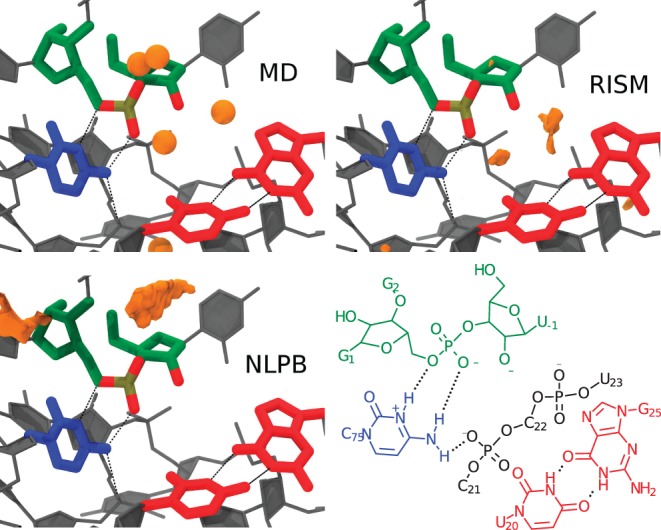
Na^+^ pair distribution functions (orange isosurfaces) computed via NLPB (*bottom left*) and 3D-RISM-PSE3 (*top right*) compared with peak positions (orange spheres) from MD and volmap (*top left*). Isosurfaces correspond to a concentration of 300 times the bulk (140 mM). Density beyond 3 Å from the active site residues was clipped for clarity.

The ultimate goal of MM/NLPB-SA and MM/3D-RISM type calculations is to assess energetics and solvation effects; however, these methods make significantly different assumptions concerning the structure of the solvent environment (e.g., standard NLPB neglects solvent–solvent correlations, whereas 3D-RISM does not). Thus, as an initial test of the quality of 3D-RISM and NLPB calculations, the HDVr-Na^+^ pair distribution functions (PDFs) were calculated on a structure from a trajectory in which a Mg^2+^ ion was not bound (Fig. 3). These PDFs describe, in an average sense, the three-dimensional distribution of Na^+^ ions around the HDVr solute without the effects of bound Mg^2+^. The ion density distribution predicted by 3D-RISM-PSE3 matches closely that from MD simulation which places a specific, buried Na^+^ ion near the nucleophile (Fig. 3). NLPB, on the other hand, does not predict this density or even qualitatively agree with either the 3D-RISM or MD simulation ion density.

As a next step, relative free energies were derived from a thermodynamic cycle (Fig. 2) describing protonation (Δ*G*_a_) and binding of Mg^2+^ at U-1:O2′ (Δ*G*_b_), as well as changes in the background concentration of Na^+^ (Δ*G*_c_). From these definitions, p*K*_a_ shifts for deprotonation of the U-1:O2′ nucleophile, Δp*K*_a_, can be defined in proportion to the difference in the free energies of deprotonation or, alternatively, the free energies of binding or change in ionic strength.

(1) $$\begin{array}{rl} \Delta \Delta G_{a}^{x} & \equiv \Delta G_{a}^{x}-\Delta G_{a}=\Delta G_{b}^{O2^{\prime -}}-\Delta G_{b}^{O2^{\prime }H}\\ & =\Delta G_{c}^{O2^{\prime -}}-\Delta G_{c}^{O2^{\prime }H}\\ & \Delta p{K_{a}}^{x}=\frac{\Delta \Delta G_{a}^{x}}{\mathrm{RT}\text{ln}10}\\ & x=Mg^{2+}\;\mathrm{or}\;Na^{+}. \end{array}$$

As shown in Table 2 (below), 3D-RISM calculations with the KH and PSE2 closures yield p*K*_a_ shifts of ∼4 units in the presence of Mg^2+^ (assumed to be explicitly bound with a 20 mM background) but only ∼2.5 units in the presence of Na^+^ (i.e., with 1 M background). The use of other repulsive–dispersive models for Mg^2+^, for both the solute and/or solvent, did not change the results by more than a few kcal/mol and never increased the magnitude of the p*K*_a_ shifts significantly beyond the reported values. Similar parameter insensitivities were observed when changing background salt concentrations (e.g., 10 mM Mg^2+^). Results for NLPB-SA displayed a trend similar to 3D-RISM, but with a much more dramatic shift in the presence of Mg^2+^ (57 units) and a shift of <1 unit in the presence of Na^+^. These results were essentially unchanged by omitting surface area terms.

Lastly, the relative free energies of the two different ionic ground states (RH and RH:Mg^2+^) can be determined as the difference in the relative free energies of deprotonation ($\Delta G_{a}^{\mathrm{Mg}2+}-\Delta G_{a}^{\mathrm{Na}+}$ in Table 2, below). Again, both RISM closures predict similar shifts of ∼2 kcal/mol in favor of the Mg^2+^ bound state. Also as above, NLPB-SA predicts a much larger shift (77.2 kcal/mol).

### QM/MM free energy surfaces

**FIGURE 4.**
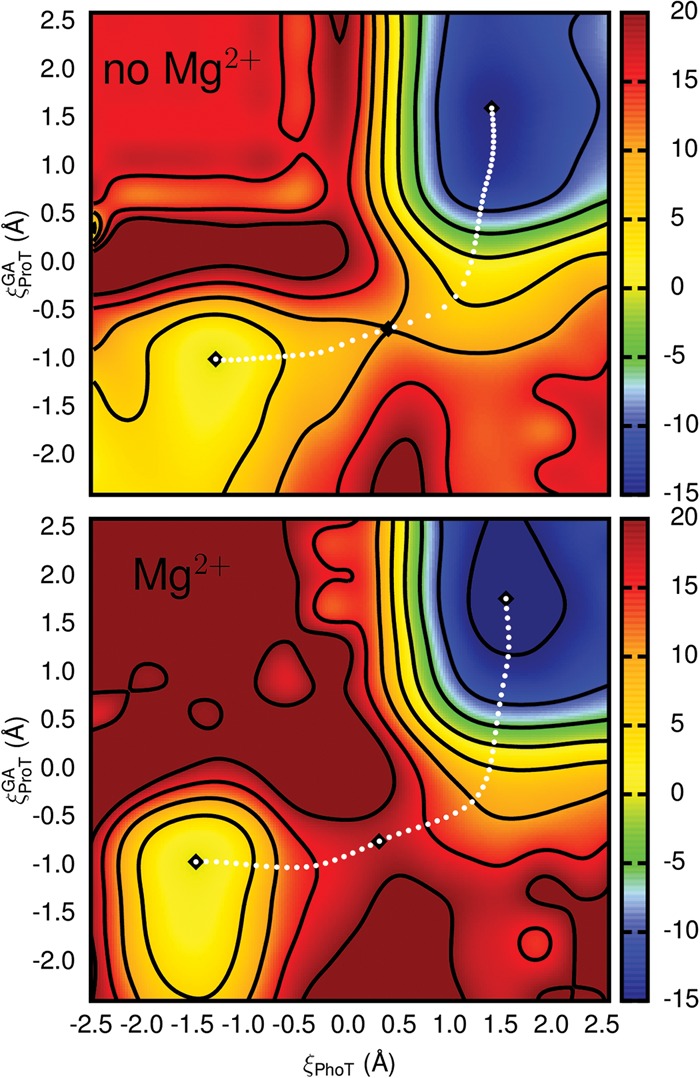
Free energy surfaces of the HDVr catalytic reaction starting from an activated (post-base) state with (*bottom*) and without (*top*) Mg^2+^ bound at the position hypothesized by Chen et al. (2010). Axis and abscissa correspond to atom transfer coordinates for general acid proton transfer and phosphoryl transfer (ξ_ProT_^GA^ and ξ_PhoT_, respectively). Free energies are in kcal/mol relative to the reactant minima with 5 kcal/mol separation of contour lines. Minima and saddle points (black diamonds) and a minimum free energy path (white dots) are also shown.

QM/MM free energy surfaces of the HDVr catalyzed reaction were calculated both with (R^−^:Mg^2+^) and without (R^−^) Mg^2+^ present in the active site (Fig. 2). Figure 4 shows the free energy surfaces defined by axes corresponding to phosphoryl transfer (ξ_PhoT_) and general acid proton transfer from C75 (ξ_ProT_^GA^). The results are largely indistinguishable, with only small differences in the location of their stationary points (Fig. 4). The shapes of the reactant and product basins are also quite similar with nearly identical eigenvalues (Table 1). This analysis can be extended further by quantifying the reaction coordinate motions in each basin. To this end, the extent of coupling between the normal mode motions was analyzed by comparing the normal mode basis to the phosphoryl/proton transfer basis. We consider the modes to be completely coupled if the phosphoryl/proton transfer component magnitudes are equal (i.e., the normal mode basis is rotated 45° with respect to the axes). This coupling can be expressed on a scale of 0–1 by calculating the absolute value of the cosine of the angle of rotation (Table 1). For both free energy surfaces the normal modes indicate significant decoupling of phosphoryl and proton transfer in the product states. In the reactant states, however, there is stronger coupling, with slow oscillation primarily along the proton transfer coordinate. The (reactive) mode orthogonal to this describes mostly high frequency phosphoryl transfer motion. The transition states are likewise indicative of strongly coupled motions, indicating that motion along the general acid coordinate increases as the reaction progresses.

**TABLE 1.**
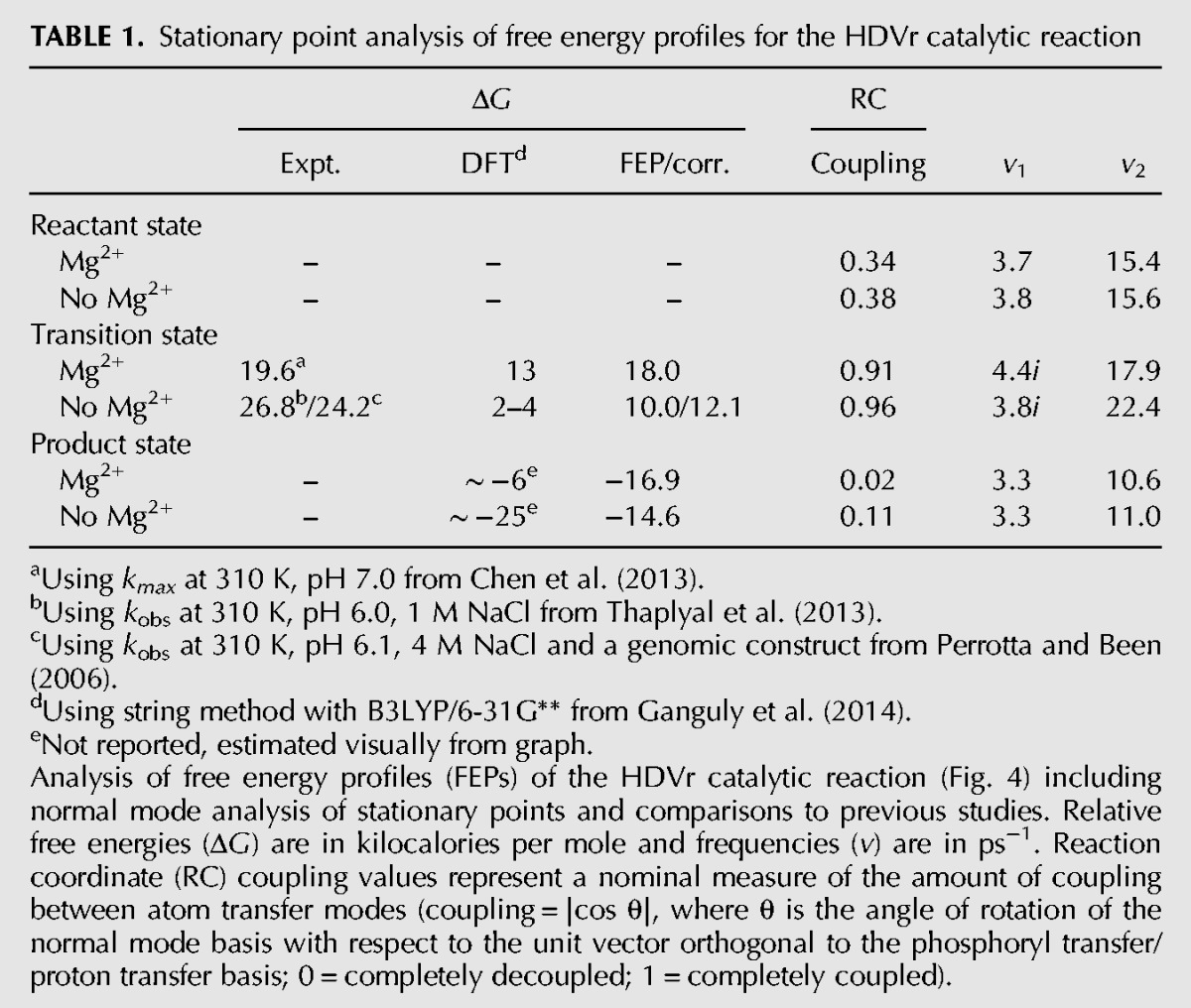
Stationary point analysis of free energy profiles for the HDVr catalytic reaction

**FIGURE 5.**
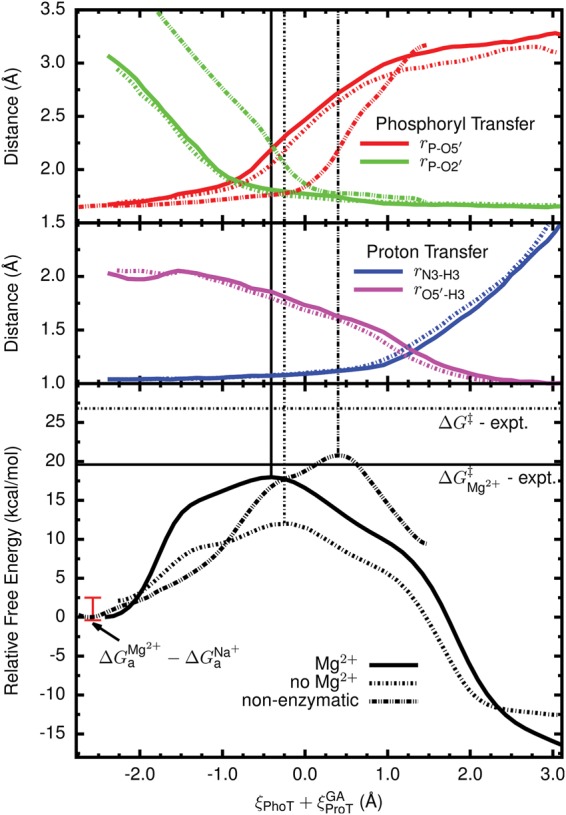
Reaction profiles (*bottom*) along the minimum free energy paths for the HDVr catalytic reaction starting from an activated (i.e., deprotonated) state with and without the presence of Mg^2+^ at the position hypothesized by Chen et al. (2010) (solid and dashed lines, respectively). The zeros of energy are set according to an estimated relative free energy of deprotonation ($\Delta G_{a}^{\mathrm{Mg}2+}-\Delta G_{a}^{\mathrm{Na}+}=-2.1\;\text{kcal/mol}$). Averages of selected bond lengths along the path are also shown (*top*). The calculated (including correction) and experimental barrier heights (from classical transition state theory, in kcal/mol) are 18.0 and 19.6 (Chen et al. 2013) and 12.1 and 26.8 (Thaplyal et al. 2013) for the reactions with and without Mg^2+^, respectively.

Analysis of the relative free energies of the stationary points can be simplified by projecting the minimum free energy paths of each surface onto a sum of atom transfer coordinates, ξ_PhoT_+ξ_ProT_^GA^ (see Materials and Methods). This leads to a simple, univariate function of the relative free energy linking the (activated) reactant and product states (Fig. 5, bottom). Calculating these paths with either the MBAR or vFEP method gave indistinguishable results within statistical error. Although the energy differences between the reactant and transition states (neglecting Jacobian corrections) are quite different with and without Mg^2+^ (18.0 and 10.0 kcal/mol, respectively), several relevant average bond lengths are nearly identical along both paths (Fig. 5, top). The only significant exception is the average length of the U-1:O5′ to G1:P bond (Fig. 5, top, red lines). Regardless, in both cases the start of the reaction is dominated by formation of the P-O2′ bond (Fig. 5, top, green lines) with an increasingly strong hydrogen bond between G1:O5′ and C75:N3 as the transition state is crossed. This hydrogen bond concertedly changes to proton transfer as the P-O5′ bond breaks (Fig. 5, top, red and magenta lines). However, the covalent bond between the proton and its donor, C75:N3, does not appear to break until the reaction has progressed considerably (Fig. 5, top, blue lines). In fact, this bond does not appear to form at all significantly until P-O5′ bond breakage has nearly completed. This is similar to the observation that the normal modes display an increasing amount of coupling between the phosphoryl and proton transfer coordinates as the reaction progresses toward the transition state.

## DISCUSSION

The main purpose of the present work is to evaluate mechanistic scenarios departing from the suggested metal ion binding mode previously proposed in the literature (Chen et al. 2010; Golden 2011) by establishing the experimentally testable consequences and identifying key aspects deserving further consideration. The computational results reported here accomplish this goal by integrating several approaches aimed at different time and length scales (i.e., fast chemical events via QM/MM MD and slower/longer length RNA motions via MM MD) as well as solvent considerations (i.e., explicit MD and 3D-RISM). The resulting new information can be used to evaluate the specific atomistic reaction pathways and allows structural/energetic analysis of the related ground states within a simple equilibrium scheme that relates ionic interactions and deprotonation of the O2′ nucleophile (Fig. 2).

### Comparison with nonenzymatic reactions

Enzymatic reactions are often best characterized by their relation to “uncatalyzed” (i.e., nonenzymatic) reactions. Calculations for several reaction models of nonenzymatic RNA cleavage were recently reported and are consistent with the computational approach used here (Radak et al. 2013a). The key difference between the enzymatic and nonenzymatic reactions is the absence of the enzymatic scaffolding (which organizes the active site) as well as the absence of a general acid (or any kind of leaving group stabilization). The nonenzymatic reaction (in this case, specific base catalyzed cleavage of an abasic dinucleotide) was also studied from a ground state containing a deprotonated nucleophile and thus is representative of the intrinsic reaction barrier and uncoupled from pH considerations.

The free energy profile of the nonenzymatic reaction is compared with those of the two HDVr catalyzed reactions (i.e., with and without Mg^2+^) in the lower part of Figure 5. Both HDVr catalyzed reactions have distinctly lower catalytic barriers and the reaction in the absence of Mg^2+^ is less than half that of the nonenzymatic reaction (10.0 versus ∼21 kcal/mol). This reduction in the barrier can be reasonably attributed to the structural and proton transfer effects already mentioned. First, the HDVr environment structurally orients the activated nucleophile in a position much closer to the scissile phosphate. Indeed, Figure 5 (top, green lines) shows that the nucleophile to phosphorous distance (*r*_P−O2′_) is ∼1 Å shorter in the reactant state of the HDVr (both metal scenarios) than for an RNA dinucleotide in solution. Moving along the free energy profile for the nonenzymatic reaction up to a distance more in line with the HDVr suggests a catalytic effect of 4–5 kcal/mol from this structural alignment alone ($\xi_{\mathrm{PhoT}}+\xi_{\mathrm{ProT}}^{\mathrm{GA}}\;\approx -1.1$Å, Fig. 5, bottom). Second, in the HDVr the protonated C75 donates two hydrogen bonds to the scissile phosphate and one of these bonds changes to a proton transfer as the reaction proceeds. An ad hoc bookkeeping of these interactions might suggest ≈2–3 kcal/mol per hydrogen bond. Along with other general electrostatic stabilization, the ∼11 kcal/mol difference between the enzymatic (without Mg^2+^) and nonenzymatic reactions can thus be reasonably accounted for.

### HDVr ground states

In estimating relative activation energies from the QM/MM free energy surfaces calculated here for the HDVr reaction, two major factors must be properly considered. First, the HDVr activated states (R^−^ and R^−^:Mg^2+^) are not identical, as they correspond to two very different ionic bound states. Second, the ground states in these simulations are assumed to be preactivated by equilibrium deprotonation of the O2′ nucleophile (see Fig. 2). For the enzymatic reaction at neutral or similar pH, however, the O2′ is protonated in the ground state; deprotonation of the nucleophile is assumed to be an equilibrium process (specific base catalysis) that governs the population of the activated reactant state. Decreasing the population of the deprotonated O2′ state in turn decreases the concentration of active ribozyme and therefore attenuates the intrinsic rate constant down to the experimentally observed value (e.g., the log-linear behavior observed in some pH-rate profiles). The population of active HDVr will depend on the correct protonation of both the O2′ and C75.

Raman crystallographic measurements have indicated that the p*K*_a_ of C75 may be anti-correlated with a Mg^2+^ binding event. However, it is possible that it is anti-correlated with a different binding site than the one investigated here (Gong et al. 2007; Chen et al. 2009). Additionally, at pH values below the p*K*_a_ of C75 (∼6), this residue will be predominantly in its active form. As such, we proceed under the assumption that C75 is protonated in all states considered here. In the present work, as in that of Ganguly et al. (2014), the deprotonation step is assumed to have already occurred before the reaction proceeds (i.e., during the QM/MM simulation). Instead, preequilibrium assumptions are used to estimate the fraction of active enzyme from the relative free energies (calculated by MM/3D-RISM) of the various unreacted states (i.e., combinations of Mg^2+^ bound/unbound and O2′ protonated/deprotonated). The combination of QM/MM and MM/3D-RISM provides a feasible approach to directly calculate the relative free energy change of the deprotonation process under different ionic conditions.

The free energy estimates from 3D-RISM generally agree in trend and magnitude (Table 2). The NLPB-SA estimates, however, do not appear to be physically realistic (the predicted p*K*_a_ shifts are far too large), nor do the Na^+^ pair distribution functions match well with those obtained from MD (Fig. 3). As such, they are not considered further. The best estimate available here is from MM/3D-RISM-PSE2, as the KH closure is known to have difficulty in predicting small molecule hydration free energies (Truchon et al. 2014) as well as excess chemical potentials for simple electrolytes (Joung et al. 2013) and excess ion distributions around DNA (Giambasu et al. 2014) (although still with greater fidelity than NLPB). It is difficult to assess the exact systematic/numerical errors of these estimates, but grid spacing effects likely will not cause errors >0.5–1 kcal/mol (Luchko et al. 2010) and systematic tests (data not shown) on the buffer size indicate errors <0.5 kcal/mol. Neglecting structural variations by clustering is probably the most substantial source of error, likely 1–2 kcal/mol.

**TABLE 2.**
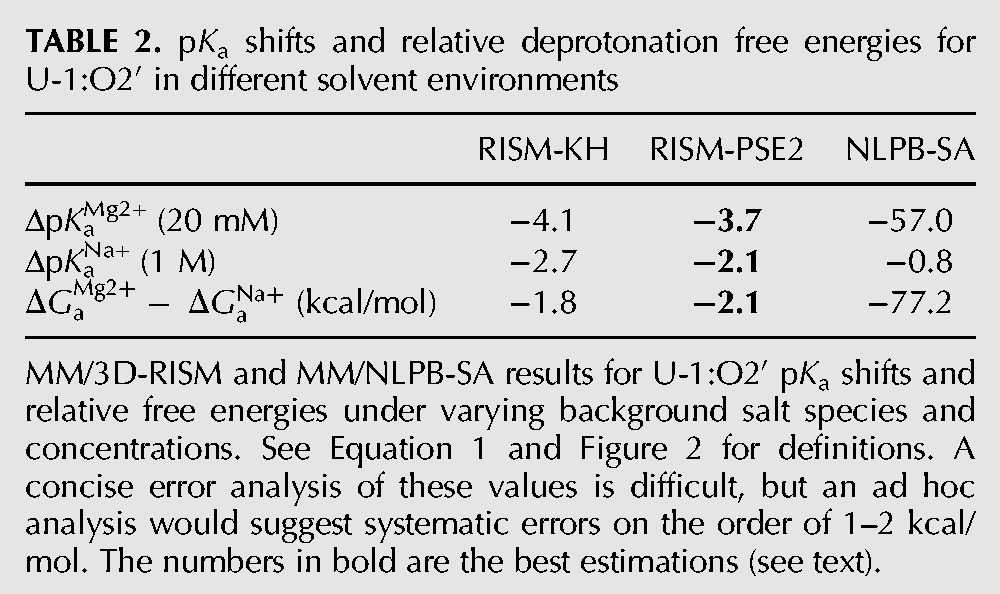
p*K*_a_ shifts and relative deprotonation free energies for U-1:O2′ in different solvent environments

### Comparison of metal-bound states

Since it is well established that the HDVr is more catalytically proficient in the presence of divalent ions than monovalent ions (Nakano et al. 2001; Perrotta and Been 2006; Thaplyal et al. 2013), it is perhaps initially surprising that the predicted free energy barrier from the activated precursor in the absence of a bound Mg^2+^ ion (10 kcal/mol) is nearly half that when Mg^2+^ is bound (18 kcal/mol, see Table 1; Fig. 5). However, as described above, the reactant state on these surfaces is a “deprotonated” oxyanion and should clearly have strong, favorable electrostatic interactions with nearby cations. The negatively charged O2′ nucleophile near Mg^2+^ is thus better stabilized by the higher concentration of proximal positive charge. This charge preferentially stabilizes the reactant state over the transition state and effectively raises the barrier. In a related observation, P–O2′ bond formation is quite advanced in both transition states (see Fig. 5), indicating that the negative charge is likely redistributed away from the nucleophile and toward the leaving group, giving rise to less favorable electrostatic interactions. The fact that the transition state structures with and without Mg^2+^ are so similar (Fig. 5, top) is sensible within a Hammond–Leffler framework since this stabilization effect should hold equally well for the charged product (note that Table 1 also shows that the “ligation” barrier is much lower in the absence of Mg^2+^). As it has been suggested that Mg^2+^ could act as the general base (Nakano et al. 2000), it may be worthwhile to examine the effect of replacing Mg^2+^ with Mg^2+^(OH^−^) in calculations that specifically examine the mechanism of nucleophile activation. Complementary to these calculations would be experimental studies that examine the kinetics of the general base step using a modified substrate with enhanced leaving group such as a 5′ thio substitution (Kath-Schorr et al. 2012) under varying ionic conditions.

In order to achieve a meaningful comparison of the reaction barriers in the presence and absence of the active site Mg^2+^, the ground states for these two reactions (RH and RH:Mg^2+^) must be thermodynamically connected. This is because the QM/MM simulations depart from precursor states (R^−^ and R^−^:Mg^2+^) that assume nucleophile activation has occurred in a preequilibrium processes. This assumption is nontrivial and the probabilities of obtaining these states (and thus the required free energy) are likely quite different. The relative shift in free energy between the two reactions can clearly be related to the free energies associated with O2′ deprotonation in both cases. Using the MM/3D-RISM framework described above, the best estimate of the free energy shift between the two profiles is ∼2.1 kcal/mol in favor of the Mg^2+^ bound mechanism (Table 2; Fig. 5). Even with rather pessimistic error assumptions, this shift is not likely to be >5–6 kcal/mol. Clearly such a shift is qualitatively in line with experiment; however, it is far short of the experimentally expected/derived value of ∼16 kcal/mol (Chen et al. 2013).

A similar finding has been reported by Ganguly et al. (2014). Nevertheless, in that work, the needed free energy correction was inferred from (1) the experimental observation of a ∼25-fold slower overall rate in the case of monovalent ions alone, (2) the measured p*K*_a_ of the nucleophile O2′ being between 1.3 and 2.7 units lower for divalent ions than for sodium ions, which can be translated as differences of between 1.8 and 3.8 kcal/mol, comparable to our MM/3D-RISM result (∼2.1 kcal/mol), and (3) the calculated free energy barriers for the self-cleavage reaction (∼13 kcal/mol and ∼3.5 kcal/mol for divalent and monovalent ions, respectively). As a result, the case with Na^+^ alone is expected to be disfavored by an additional ∼8 to 10 kcal/mol. Hence our study, where a theoretic correction of the free energy shift is calculated, and the study by Ganguly et al. (2014), where the needed free energy correction is inferred from experimental measurements and calculated energy barriers, both conclude that, compared with the experimental measurements, the current structural model favors the case with Na^+^ alone by ∼8 to 10 kcal/mol.

To summarize, the preceding analysis is only partially aligned with existing experimental interpretations. The reaction barrier for the HDVr with a preactivated nucleophile and site-bound Mg^2+^ is closely aligned with values derived from experiment, more so than previous estimates by Ganguly et al. (2014) (Table 1). Note that this agreement requires the assumption that this bound ion is present nearly 100% of the time, which is not implausible considering the low saturation point (∼10 mM Mg^2+^) of several metal titration experiments (Nakano et al. 2000, 2001; Chen et al. 2009, 2013). Combining this assertion with the ∼4 unit downward p*K*_a_ shift of the O2′ nucleophile predicted here (in 20 mM Mg^2+^) makes this a cohesive scenario.

However, when Mg^2+^ is substituted with a large Na^+^ concentration (1 M), the predicted variation is not as dramatic as that observed experimentally. This could be for a variety of reasons. First, the core structure obtained in the simulations, while slightly different than that obtained crystallographically, is heavily biased toward a folded state facilitated by Mg^2+^. The inline conformation needed for the reaction seems unaffected by the ionic environment both in our calculations and in the work by Ganguly et al. (2014). Although it has been suggested that the overall HDVr fold will not be altered when only monovalent ions are present (Ke et al. 2007), the local conformation could be altered upon charge/protonation state changes. As shown recently, the HDVr inline conformation highly correlates with the protonation state of C75 (Sripathi et al. 2014). Although a low Na^+^ concentration (∼140 mM) was able to stabilize this structure here, the present simulations cannot confirm that it is plausible for this structure to form in the complete absence of divalent metal ions in the first place. Second, the structure used here assumes constant protonation of C75. The justification here again derives from an overall fold obtained in the presence of Mg^2+^. This is problematic if the p*K*_a_ shift experienced by C75 is assumed to originate from the fold of the active site. In addition, both MD and 3D-RISM predict a substantially higher density of Na^+^ (as many as three distinct sites) than Mg^2+^ (one to two sites) near C75 when the salt environment is changed. The overly favorable p*K*_a_ shift in the presence of molar Na^+^ predicted here may therefore be more reflective of the RNA fold rather than salt interactions. This interpretation should imply a correlation between folding due to ionic interactions and active site organization arising from protonation of C75, yet most experimental results indicate an “anti-correlation” of these two factors (Gong et al. 2007) (an exception to this is Mg^2+^ driven formation of a base triplet involving protonated C41 [Nakano and Bevilacqua 2007]). It may be possible to probe these interactions further through judicious use of inactive C75U mutants which, at the time of this writing, have been rescued by imidazole titration (Perrotta et al. 1999; Thaplyal et al. 2013) and leaving group activation (Das and Piccirilli 2005), but never in conjunction with a high monovalent salt concentration. A similar C75Δ mutant has recently been rescued by imidazole in conjunction with ammonium ions in the absence of Mg^2+^ (Thaplyal et al. 2015). This suggests a more active role for NH_4_^ +^ compared with other monovalent ions, an idea which was also probed by kinetics studies of thio substituted wild-type-like constructs (Thaplyal et al. 2015). Furthermore, a recent refitting of crystallographic data for a C75U mutant *does* show a fold similar to that here, but lacks both an inline attack angle for U-1:O2′ as well as proper alignment of the leaving group and the (missing) general acid (Kapral et al. 2014). Hence, further work is warranted in order to explore the interplay between C75 protonation, enzyme folding, and ion binding in different mutants and under different ionic conditions.

### Implication of the reaction pathway

A key aspect of the mechanistic model investigated here is the high similarity between the reaction pathways with and without Mg^2+^ present. If these pathways are in fact similar, then a reasonable, zeroth order approximation would suggest that their heavy atom (^18^O) kinetic isotope effect (KIE) signatures would be similar. The present calculations are not sufficiently detailed to make quantitative predictions as to what the magnitude of these effects would be. The mechanism described here, however, makes specific predictions regarding the details of transition state bonding and the qualitative impact of this on observed KIEs can be predicted. Likewise, alternative mechanisms recently described by Ganguly et al. (2014) for the Mg^2+^ and Na^+^ reactions also make implicit predictions regarding the likely magnitude of isotope substitution on the reaction rate constant. A key feature of the mechanisms in both their work and ours is the asynchronicity (although to different extents) of P–O5′ bond cleavage, which lags behind P–O2′ bond formation and leads to an associative transition state. Such a transition state takes the form of a phosphorane with significant charge accumulation on the nonbridging oxygens. Relevant O2′ and O5′ KIE data would be useful evidence to interpret this transition state model. Furthermore, protonation from C75:N3 appears to occur late along the reaction coordinate. This feature is readily apparent in Figure 5 by noting the position with respect to the transition state where the lengths of the forming and cleaving bonds cross. The scenario here predicts that there would be minimal contribution to the O5′ KIE from the formation of the new O5′-H bond. In contrast, in Ganguly et al. (2014) this point is nearly coincident with the transition state. Obviously, precise O5′ KIE measurements would be able to clarify this discrepancy.

### Further aspects

The present work investigated one distinct Mg^2+^ binding mode suggested based on crystallographic data and this mode appears to be consistent with a large amount of structural, biochemical, and computational data. However, it cannot be excluded that other binding modes may also be consistent with these data and give similar results within the present thermodynamic framework. Nonetheless, a key characteristic of the mechanism described here is that it involves multiple compensating effects due to the presence of Mg^2+^. The protonated ground state provides a favorable binding position which gives rise to a p*K*_a_ shift of the nucleophile toward neutrality. This promotion of the activated precursor state is necessarily offset by stabilization of the reactant over the transition state, as the ion provides a +2 charge while the general acid provides only +1. That is, in order for the system to progress toward the transition state, electrons must migrate to a region with “lower” positive charge. This penalty would of course be lower if the metal ion were absent, but then the nucleophile would be much less likely to be found in an activated state and thus the increased electrostatic barrier is unavoidable. This effect becomes obvious when the Mg^2+^ ion is replaced by more diffusely held Na^+^ ions and the barrier is lowered, but at the expense of a slightly less populated precursor state. If this reasoning is correct, then other chemical modifications to the system which lower the O2′ p*K*_a_ should enhance the cleavage rate in low concentrations of Mg^2+^ or even rescue the reaction in the presence of monovalent ions alone. Such modifications might likely include the fluoromethyl substitutions made by Ye et al. (2007) at the C2′ position of a chimeric RNA oligomer.

## CONCLUSION

Molecular simulations provide a convenient and robust framework for analyzing mechanistic pathways, especially those pertaining to complex biocatalytic systems. The key strength of such methods is that they provide unambiguous atomistic detail that can be mapped to experimental observables. In the present work, we have performed simulations of two hypothetical reaction channels in the HDVr in order to critically assess a recently proposed mechanistic model involving a site-bound Mg^2+^ as well as an alternative Na^+^ bound scenario. Once the results are properly contextualized within the required thermodynamic assumptions of metal ion binding and the resulting p*K*_a_ shift of the nucleophile, they only partially agree with the experimental data, although the reasons for this appear to follow rather directly from chemical intuition. The results are consistent with a mechanistic model whereby an active site metal Mg^2+^ ion facilitates nucleophile activation and C75 acts as a general acid catalyst. However, the results for a highly analogous scenario involving Na^+^ in the same role are exceedingly at odds with known experimental results. This study represents a distinct advance in that the results are integrated in a well-defined thermodynamic framework. The specific structural and dynamical details of this mechanism suggest several areas where additional experiments could probe this model further, especially the measurement of KIEs. In addition, a hitherto neglected thio substitution at U23 and chemical modification of the U-1:O2′ p*K*_a_ could provide evidence that would require amendment of this mechanistic model in order to maintain consistency.

## MATERIALS AND METHODS

### Molecular dynamics

The initial model structure coordinates (see Golden 2011) were used to generate multiple solvated topologies with roughly identical atomic compositions. These systems all included protonation at C75 and C41, the two crystallographic Mg^2+^ ions nearest the active site, ∼140 mM NaCl and enough Na^+^ ions to neutralize the RNA net charge. For simulations in which Mg^2+^ was not desired to be in the active site, that ion was swapped with a bulk Na^+^ and the system was then extensively reequilibrated.

MM and QM/MM MD simulations were performed using the AMBER 14 (Salomon-Ferrer et al. 2013) suite of programs. Atoms in the MM region were treated with the AMBER FF10 force field (Cornell et al. 1995; Wang et al. 2000; Pérez et al. 2007; Zgarbová et al. 2011) while those in the QM region were described by the AM1/d-PhoT semi-empirical Hamiltonian (Nam et al. 2007). The solvent environment was modeled using the TIP4P-Ew (Horn et al. 2004) rigid water model and the associated alkali metal and halide ion parameters of Joung and Cheatham (2008); magnesium was modeled based on calculations by Mayaan et al. (2004). Long-range electrostatics were treated using periodic boundary conditions and the particle mesh Ewald method (Essmann et al. 1995; Walker et al. 2008).

Long MD trajectories (>900 nsec in total) were propagated from multiple initial states in which U-1:O2′ was either neutral or deprotonated and a Mg^2+^ ion was or was not specifically bound at the active site. C75:N3 and C41:N3 were always protonated, as in previous works (Lee et al. 2011; Veeraraghavan et al. 2011a,b; Chen et al. 2013). In the case in which Mg^2+^ was not bound, the ion was replaced with a Na^+^ ion from the bulk and the system was reequilibrated, providing a gradual transition toward the new ion environment. Trajectories were structurally analyzed for use in 3D-RISM and nonlinear Poisson–Boltzmann-surface area (NLPB-SA) calculations and were also used as starting structures for QM/MM trajectories.

### 3D-RISM and NLPB calculations

3D-RISM and NLPB-SA calculations were performed with AMBER (Luchko et al. 2010) and its interface to the Adaptive Poisson–Boltzmann Solver (APBS) (Baker et al. 2001; Konecny et al. 2012). Multiple RISM closures, including the Kovalenko–Hirata (KH) (Kovalenko and Hirata 1999) and *n*th order partial series expansion (PSE*n*, *n* = 2,3) (Kast and Kloss 2008) closures, were compared. Solvent density and solvation free energy calculations were performed on structures derived from long MD simulations by removing all solvent atoms (except bound Mg^2+^, when appropriate) and, when applicable, changing the charge vector to that of the deprotonated nucleophile. Relative free energies were then estimated in the usual way from the solvation free energies (Genheden et al. 2010).

The structures for 3D-RISM and NLPB-SA calculations were derived as follows. Structural analysis first was performed on MD trajectories via a hierarchical agglomerative clustering algorithm (as implemented in CPPTRAJ [Roe and Cheatham 2013]) using the mass-weighted root mean square deviation (RMSD) of heavy atoms in the active site, defined as residues U-1, G1, U20, C21, C22, U23, C24, G25, and C75. Because the U-1 nucleobase is solvent exposed it is free to undergo *syn*/*anti* inversion which causes large changes in the RMSD while the rest of the active site remains constant. Accordingly, all nucleobase heavy atoms from U-1 were omitted from RMSD calculations. For each trajectory the cluster count (a required input parameter) was sequentially reduced from six until no more than one cluster had a fractional occupation <1%. In all cases this procedure gave rise to primary clusters with >80% occupancy. The centroid of these clusters (i.e., the single most representative simulation snapshot by the criteria used) was then used for all free energy calculations.

RISM solvent–solvent susceptibilities were obtained via DRISM (Perkyns and Pettitt 1992) (65536 grid points, 0.0125 Å spacing). The temperature and dielectric constant were set to 300 K and 78.4461950541166, respectively. The solvent was modeled with the cSPC/E water model (Luchko et al. 2010) and ion parameter choices consistent with the MD protocol. A constant density approximation was assumed in which the density of water sites was lowered from the net density of 55.428 M (≈0.0334 molecules/Å^3^) as the salt concentration increased. 3D-RISM calculations used a 96 Å buffer distance and no solute–solvent interaction cutoff (“--solvcut” ≫ 96). The residual was solved to a tolerance of 10^−6^.

Because NLPB calculations are so widespread in the literature, especially on RNA systems (e.g., the work of Chen et al. [2013]; Misra and Draper [2001]; Veeraraghavan et al. [2011a]), a slightly different protocol from 3D-RISM was used in order to make our results comparable to previous NLPB studies. Following Misra and Draper (2001), dielectric constants of 80.0 and 2.0 were used for the solvent and solute interior, respectively. Solute atom radii were taken from the default mbondi set in AmberTools 14. The only exception to this were explicit Mg^2+^ ions, which were given a radius of 1.45 Å; again, see Misra and Draper (2001). Implicit Na^+^ and Cl^−^ ions were modeled with an exclusion radius of 2.0 Å. Fine grid lengths and spacings were chosen as close as possible to those from 3D-RISM while still satisfying the multigrid constraints imposed by APBS (Baker et al. 2001). Coarse grid lengths used the same number of grid points but twice the box length (i.e., half the fine grid resolution).

### QM/MM Hamiltonian replica exchange

QM/MM Hamiltonian replica exchange umbrella sampling simulations were performed using a novel asynchronous protocol (Radak et al. 2013b) with exchanges attempted at 5-psec intervals. Sampling in each replica averaged 60–100 psec (235 nsec total). The replica states (>1300 per free energy surface) were defined by harmonic restraints, *U*(ξ), on two atom transfer coordinates:

(2) $$U(\xi )=k{\left(\xi -\xi_{0}\right)}^{2}\;\xi \equiv r_{X-Y}-r_{Y-Z}.$$

Here *r*_*X*__−*Y*_ indicates the distance between atoms X and Y. For phosphoryl transfer, ξ_PhoT_, *X* = G1:O5′, *Y* = G1:P, *Z* = U-1:O2′, and *k* = 50 kcal/mol-Å^2^ and for proton transfer of the general acid, $\xi_{\mathrm{ProT}}^{\mathrm{GA}}$, *X* = G:O5′, *Y* = C75:H3, *Z* = C75:N3, and *k* = 60 kcal/mol-Å^2^ (C75:N3 is assumed to be protonated). The restraint locations, ξ_0_, were never more than 0.15 Å apart. The resulting data were analyzed by the multistate Bennet acceptance ratio (MBAR) (Shirts and Chodera 2008) in tandem with a Gaussian kernel density estimator (see Radak et al. 2013a for details) as well as the recently developed variational free energy profile (vFEP) method (Lee et al. 2014).

A key advantage of the variational free energy profile method is that it readily constructs smooth, differentiable free energy surfaces. It is thus straightforward to identify and assess stationary points via normal mode analysis provided that a mass can be meaningfully assigned to the relevant coordinates. In the present case the progress coordinates are taken to be atom transfer coordinates approximated as the asymmetric stretching modes of the appropriate collinear triatomic. For simplicity, the reduced masses were calculated using force constants from the AMBER force field (Cornell et al. 1995; Wang et al. 2000; Pérez et al. 2007; Zgarbová et al. 2011). Large changes in the parameters (up to 100%) changed the masses by <1%. The mass matrix so obtained was then used to mass weight the Hessian at each stationary point and yielded the desired eigenvalues after appropriate unit conversions. The accompanying eigenvectors were then used to calculate the reaction coordinate coupling values in Table 1 by taking the absolute value of the dot product with the unit vector having a 45° angle with respect to the axes.
